# Supplementary material for: Systemic Mobilization of Breast Cancer Resistance Protein in Response to Oncogenic Stress
Source: Cancers (Basel). 2022 Jan 9;14(2):313. doi: 10.3390/cancers14020313 (PMC8773772; doi:10.3390/cancers14020313)
Supplement: Supplementary file 1 [file cancers-14-00313-s001.zip › cancers-1482649-SI.pdf]

Supplementary Materials: Systemic Mobilization of Breast Cancer Resistance Protein in Response to Oncogenic Stress

Małgorzata Szczygieł , Marcin Markiewicz, Milena Julia Szafraniec, Agnieszka Hojda, Leszek Fiedor and Krystyna Urbanska

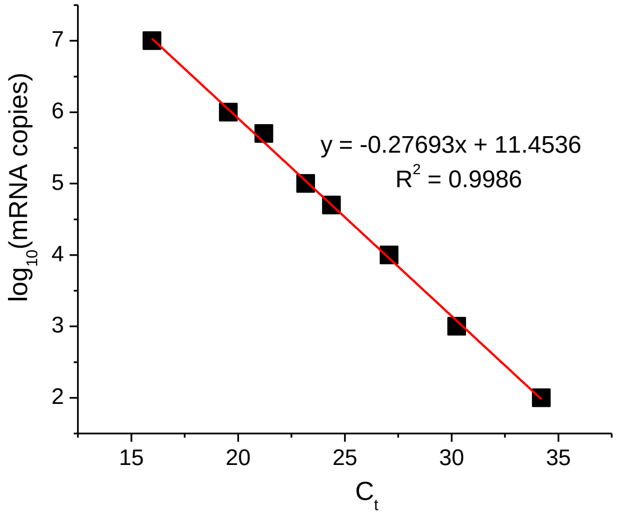

Figure S1. A calibration curve for mRNA quantification. Several dilutions of a synthetic oligonucleotide complementary to the Taq-man probe were used to generate the curve. The resulting Ct values are plotted as a function of the decimal logarithm of the template amounts and a linear trend is fit to the data.
